# 8-*epi*-Salvinorin B: crystal structure and affinity at the κ opioid receptor

**DOI:** 10.1186/1860-5397-3-1

**Published:** 2007-01-09

**Authors:** Thomas A Munro, Katharine K Duncan, Richard J Staples, Wei Xu, Lee-Yuan Liu-Chen, Cécile Béguin, William A Carlezon, Bruce M Cohen

**Affiliations:** 1 Mailman Research Center, McLean Hospital, 115 Mill St, Belmont MA 02478-9106, USA; 2 Department of Chemistry and Chemical Biology, Harvard University, Cambridge MA 02138, USA; 3 Department of Pharmacology, Temple University, 3420 N. Broad Street, Philadelphia, PA 19140, USA

## Abstract

There have been many reports of epimerization of salvinorins at C-8 under basic conditions, but little evidence has been presented to establish the structure of these compounds. We report here the first crystal structure of an 8-*epi*-salvinorin or derivative: the title compound, **2b**. The lactone adopts a boat conformation with the furan equatorial. Several lines of evidence suggest that epimerization proceeds via enolization of the lactone rather than a previously proposed indirect mechanism. Consistent with the general trend in related compounds, the title compound showed lower affinity at the kappa opioid receptor than the natural epimer salvinorin B (**2a**). The related 8-*epi*-acid **4b** showed no affinity.

## Introduction

Salvinorin A (**1a**), isolated from the hallucinogenic sage *Salvia divinorum*,[1] is a potent and selective κ opioid receptor (KOR) agonist.[2] Because it is the first known non-nitrogenous compound to have biologically significant actions at mammalian opioid receptors, **1a** enables new approaches to studies of endogenous opioid receptor systems. KOR ligands, in particular, have attracted considerable interest because of their effects on mood states.[3–6] Recently, numerous synthetic derivatives of **1a** have been prepared and evaluated for activity at opioid receptors. Some potent agonists have been identified which are expected to show increased stability or solubility.[7] Others have increased affinity and potency, [8] or altered subtype selectivity.[9] As yet, however, no derivatives of **1a** appear to be KOR partial agonists or antagonists, classes of agents that may have utility in the treatment of psychiatric conditions such as depression or mania.[4–5,10]


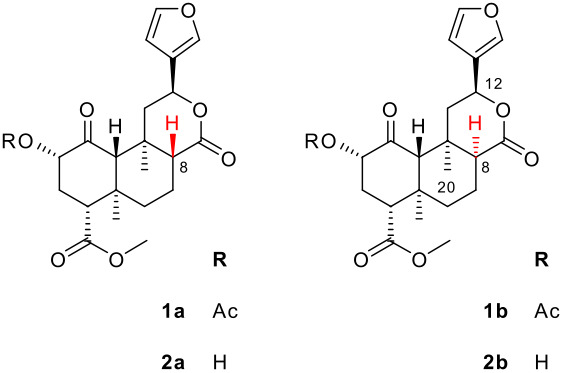


Salvinorins tend to isomerize under basic conditions. Valdés reported that borohydride reduction of **1a** gave an unidentified stereoisomeric byproduct, which could be converted to an undetermined stereoisomer of **1a**.[11] The latter compound was subsequently identified by Brown as 8-*epi*-salvinorin A (**1b**).[12] Brown also reported that deacetylation of **1a** under basic conditions gave 8-*epi*-salvinorin B (**2b**), but did not characterize either compound. Several further reports of epimerization at C-8 appeared over the following decade, [13–14] but no characterization data was presented. Valdés later identified the byproduct mentioned above as 8-*epi*-diol **3**.[15] Characterization data was given, but the basis of the structure assignment was not stated.


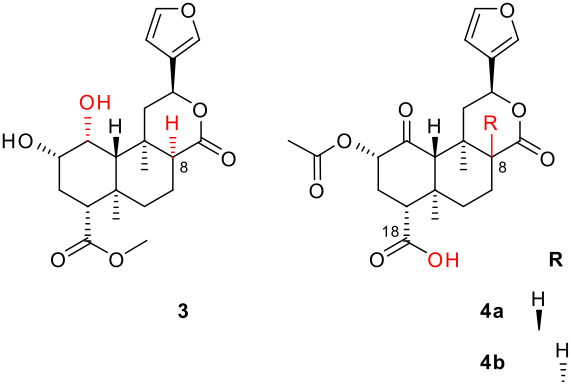


The first structure elucidation of one of these compounds was of 8-*epi*-salvinorin A (**1b**).[16] The *trans*-diaxial H-8 coupling constant found in **1a** was absent in **1b**, establishing an equatorial configuration. Also, irradiation of H-12 in **1b** gave a strong nOe enhancement of H-8. The corresponding experiment on **1a** gave instead an enhancement of H-20. These findings can be extrapolated to **2b**, since acetylation gives **1b** quantitatively.[9,17] Conflicting ^1^H NMR data for **2b** itself were later reported by two groups.[8–9] The ^1^H NMR spectrum of **2b** is reproduced in Supporting Information File 1; the corresponding amended data have been reported previously.[17] Interestingly, epimerization has also recently been reported under acidic conditions.[18]

The epimers can be readily identified by TLC: the unnatural compounds almost invariably spot above the natural compounds in EtOAc/hexanes, and give a blue rather than pink/purple colour when visualized with vanillin.[19] The unnatural epimers are also recognizable by their distinctive H-12 multiplet in ^1^H NMR, which resembles a broad doublet shifted upfield to ~δ 5.30 ppm. Many 8-*epi*-salvinorin derivatives have now been reported, although many have not been fully characterized.[7–9,17–18,20–24] Thus, the many reports of 8-*epi*-salvinorins and derivatives have been based on limited data.

## Results and Discussion

**Figure 1:**
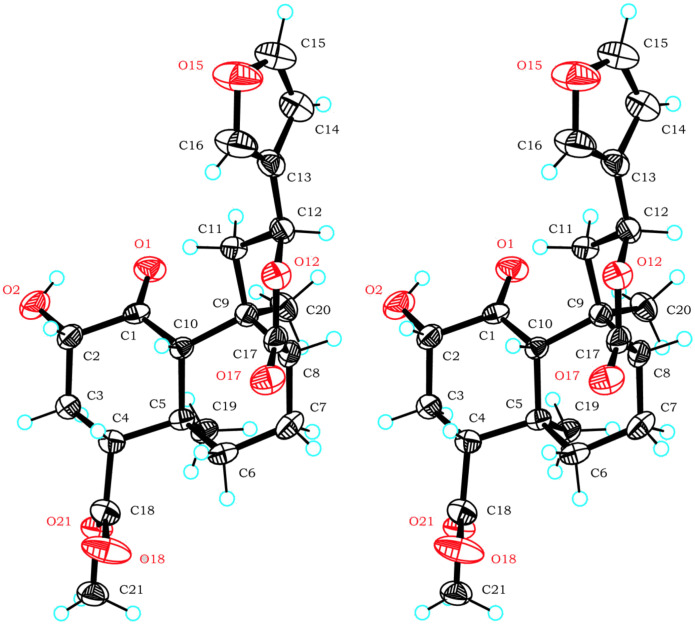
Stereoview of the molecular structure of **2b**, showing 50% probability displacement ellipsoids and the atom-numbering scheme. Only one of the two molecules in the asymmetric unit is shown.

**Figure 2:**
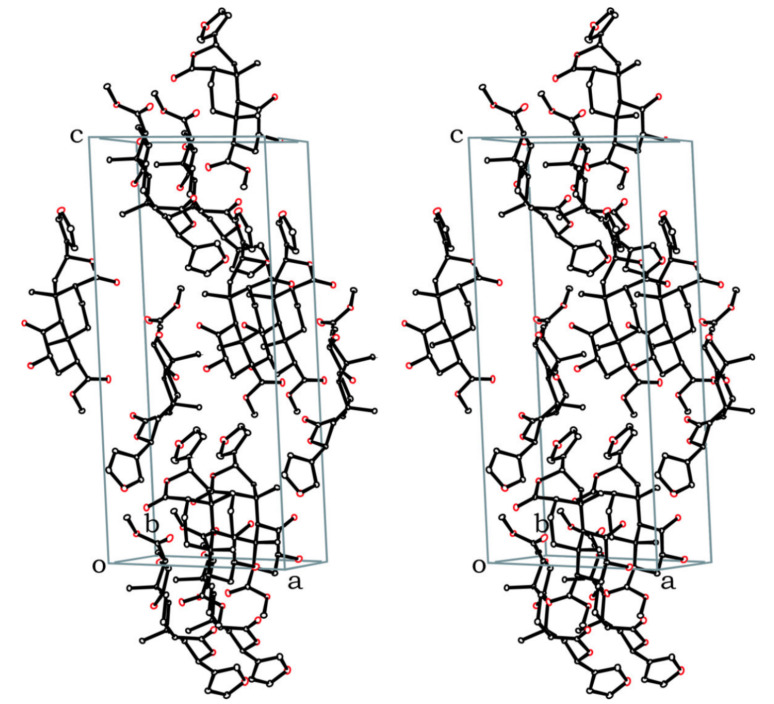
Stereoview of the packing of **2b**. H atoms are not shown.

The crystal structure presented here (Figure 1) is the first reported for an 8-*epi*-salvinorin or derivative. It firmly establishes the structure of **2b**, and therefore of **1b**. The lactone carbonyl C-17 is axial with respect to the B ring (C6-7-8-17 torsion angle 77° versus 173° in **1a**).[1] The lactone itself adopts a boat conformation with the furan equatorial (C9-11-12-13 torsion angle 179°). This is as predicted in solution, on the basis of a *trans*-diaxial coupling constant for H-12.[17] This is also consistent with the crystal structures of furanolactones with all other possible C8/9/12 stereochemistries (*trans/anti*, *trans/syn* and *cis/syn*) – the furan is equatorial in all cases.[17] The rest of the structure is very similar to the crystal structure of **1a**.[1] The hydroxyl group participates in an intramolecular hydrogen bond with the ketone (O2-H2···O1, 2.12 Å). There are no intermolecular hydrogen bonds. The asymmetric unit consists of two molecules; the only substantial difference between them is in the rotation of the furan ring (C11-12-13-14 torsion angle -87° (A) versus 53° (B)). The crystals are monoclinic, space group *P*2_1_ (see Figure 2). The crystallographic data can be found in Supporting Information File 2; the structure factors are in Supporting Information File 3. The crystallographic data have also been deposited with the Cambridge Crystallographic Data Centre (CCDC 626179).[25] 8-*epi*-Salvinorins and derivatives have a much weaker tendency to crystallize than their natural counterparts. Unsurprisingly, therefore, **2b** has a lower melting point (192–196°C) than **2a** (239–240°C).[17]

Configuration at C-8 is biologically significant. The affinity and potency of 8-*epi*-salvinorin A (**1b**) at the KOR are dramatically lower than those of **1a**.[16] This finding has been replicated several times.[8–9,20] The same trend is evident with many salvinorin derivatives: epimerization of active compounds at C-8 reduces affinity and potency.[8–9,20,23–24] Very few exceptions to this trend have been reported to date.[8,23] These include 8-*epi*-salvinorin B (**2b**) itself, whose binding affinity (*K*_i_ = 43 nM) was reportedly greater than that of the natural epimer **2a** (111 nM).[8] To explore this anomaly, we submitted a new sample of **2b** for *in vitro* testing at the KOR. Binding affinity, potency and efficacy were determined as previously described (Table 1).[26]

**Table 1:** Affinities (*K*_i_), potencies (EC_50_), and efficacies at the KOR.

Compound	*K*_i_ ± SEM ^a,b^	EC_50_ ± SEM^b,c^	*E*_max_ ± SEM^d^
	nM	nM	%

**1a**	2.4 ± 0.4	1.8 ± 0.5	98 ± 3
**2b**	304 ± 46	214 ± 33	90 ± 2
**4a**	>10,000	-	-
**4b**	>10,000	-	-
U50,488H	2.2 ± 0.3	1.4 ± 0.3	100

^a^Inhibition of [^3^H]diprenorphine binding to membranes of Chinese hamster ovary cells stably transfected with the human KOR (CHO-hKOR). ^b^Mean ± SEM of three independent experiments performed in duplicate. ^c^Enhancement of [^35^S]GTPγS binding to CHO-hKOR membranes. ^d^Relative to that of U50,488H control.

The binding affinity of **2b** (*K*_i_ = 304 nM) was lower than those previously reported for salvinorin B (**2a**) under the same conditions (66, 111 or 155 nM).[7–8,27] An early report that **2a** was inactive employed a different radiolabeled ligand, [^3^H]bremazocine.[28] Subsequent testing with [^3^H]diprenorphine by the same group gave concordant values for the relative affinity of **2a**.[17] Thus, our data suggest that **2b** in fact has a lower affinity than **2a**, consistent with the general trend mentioned above. We also reexamined the epimeric acids **4**.[16] In a previous report, **4a** was found to be inactive (*K*_i_ > 1,000 nM), but the 8-epimer **4b** showed high affinity at the KOR (49 nM).[23] In contrast, our current samples of both **4a** and **4b** showed no affinity at the KOR (Table 1).

Given the very high binding affinity of **1a**, contamination of an inactive or weakly active compound with even traces of **1a** will cause large errors. Flash chromatography in EtOAc/hexanes effectively separates **2b** from **2a**, but not from **1a**. To overcome this, we re-chromatographed our sample in acetone/CH_2_Cl_2_, which resolves **2b** from **1a**, and verified purity by ^1^H NMR [Supporting Information File 1]. No methoxy peak corresponding to **1a** (δ 3.72) was apparent above baseline noise. We separated **4a** and **4b** with difficulty by repeated chromatography in EtOAc/hexanes. The sample of **4a** contained traces of an inseparable impurity, which if active might artificially elevate the apparent binding affinity. Since the sample showed no affinity, however, this problem does not arise. The ^1^H NMR spectra are reproduced in Supporting Information File 1.

**Scheme 1:**
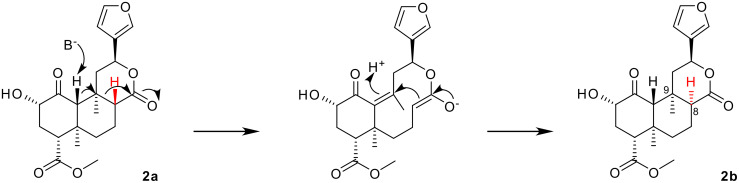
Koreeda *et al's* proposed mechanism for the epimerization.

There is no consensus on the mechanism of base-catalyzed epimerization at C-8. Koreeda and coworkers proposed a complex mechanism, initiated by ketone enolate formation. The configuration of H-8 is inverted indirectly, without exchange, by cleavage of the C-8/9 bond (see Scheme 1). [11–13] The simpler mechanism of enolization of the lactone itself has also been proposed.[16] A detailed case for this mechanism has been presented, giving evidence that H-8 exchanges under mildly basic conditions, and that similar furanolactones lacking the ketone also undergo epimerization.[17] Other workers remain undecided.[8,18]

## Supporting Information

File 1 Experimental details; statement of author contributions; ^1^H NMR spectra of **2b, 4a** and **4b** (Portable Document Format).

File 2 Crystal structure of **2b** (Crystallographic Information File).

File 3 Structure factors for **2b** (Crystallographic Information File).
