# Supplementary material for: 8-epi-Salvinorin B: crystal structure and affinity at the κ opioid receptor
Source: Beilstein J Org Chem. 2007 Jan 9;3:1. doi: 10.1186/1860-5397-3-1 (PMC1805503; doi:10.1186/1860-5397-3-1)
Supplement: File 1 — Experimental details; statement of author contributions; 1H NMR spectra of 2b, 4a and 4b (Portable Document Format). [file Beilstein_J_Org_Chem-03-01-s001.pdf]

8-*epi*-Salvinorin B: crystal structure and affinity at the κ opioid receptor

Thomas A. Munro, Katharine K. Duncan, Richard J. Staples, Wei Xu, Lee-Yuan Liu-Chen, Cécile Béguin, William A. Carlezon, Jr. and Bruce M. Cohen.

Additional data file 1 – Experimental section and ^{1}H NMR spectra

Table of contents

	Page
Experimental section	1
Statement of author contributions	2
References	3
^{1}H NMR spectra: **2b**	4
**4a**	5
**4b**	6

Experimental

X-ray diffraction analysis of ***2b***. Data was collected using a Bruker SMART CCD diffractometer with an Oxford low-temperature apparatus (193 K), using Mo Kα radiation and a graphite monochromator. A suitable crystal was chosen and mounted on a glass fiber using grease. Data were measured using omega scans of 0.3° per frame for 30 s, such that a hemisphere was collected. A total of 1271 frames were collected with a final resolution of 0.76 Å. The first 50 frames were recollected at the end of data collection to monitor for decay. *Structure refinement.* Software used for data collection: ASTRO.[1] Cell refinement: SMART.[2] Data reduction: SAINT.[3] Structure solution: SHELXS-97.[4] Structure refinement: SHELXL97.[5] Molecular graphics: ORTEP-3.[6] Preparation of CIF for publication: XCIF.[7] The structures were solved by the direct method and refined by the least squares method on F^2. All H atoms were placed in calculated positions and refined using a riding model. The Flack parameter obtained was inconclusive; in order to use all data collected, Friedel pairs were not merged. The absolute stereochemistry shown for **2b** is taken from that of **1b**, which has been verified by exciton chirality circular dichroism [8] and crystal structures of halogenated derivatives.[9,10]

Radioligand binding assays. Affinities were determined, as previously described, by competitive inhibition of [^{3}H]diprenorphine binding to membranes prepared from Chinese hamster ovary cells stably transfected with the human KOR.[11]

Preparation of ***2b***. Salvinorin A (**1a**), isolated from *S. divinorum* as previously described,[12] was treated with $NaHCO_3$ in MeOH, giving **1b** after trituration in MeOH.[13] The supernatant was evaporated and purified by flash column chromatography on silica gel (33% EtOAc/hexanes) to give **2b** as a colorless resin. Evaporation from EtOAc at room temperature in a fume hood gave colorless blades

(m.p. 192-196 °C). Other characterization data (TLC, ^{1}H and ^{13}C NMR, IR, optical rotation and HRMS) have been reported previously.[14]
InChI=1/C21H26O7/c1-20-6-4-12-19(25)28-15(11-5-7-27-10-11)9-21(12,2)17(20)16(23)14(22)8-13(20)18(24)26-3/h5,7,10,12-15,17,22H,4,6,8-9H2,1-H3/t12-,13+,14+,15+,17+,20+,21+/m1/s1

*Preparation of **4a** and **4b**.* Treatment of **1a** with LiI in refluxing pyridine [15] and exhaustive (10 ×) flash column chromatography on silica gel (66 -100% EtOAc/hexanes, followed by 1% AcOH/10% MeOH/EtOAc) gave **4b**, followed by **4a**. The ^{13}C NMR data matched those reported previously for the opposite epimers;[15] ^{1}H NMR data differed considerably.

***O*-Demethylsalvinorin A (4a).** InChI=1/C22H26O8/c1-11(23)29-15-8-14(19(25)26)21(2)6-4-13-20(27)30-16(12-5-7-28-10-12)9-22(13,3)18(21)17(15)24/h5,7,10,13-16,18H,4,6,8-9H2,1-3H3,(H,25,26)/t13-,14-,15-,16-,18-,21-,22-/m0/s1/f/h25H
TLC (EtOAc): hR_f = 24, purple/pink to vanillin/H_2SO_4;
^{1}H NMR (CDCl$_3$): δ 7.41 (1H, dt, *J* = 1.8, 0.8 Hz), 7.39 (1H, t, *J* = 1.8 Hz), 6.38 (1H, dd, *J* = 1.8, 0.9 Hz), 5.54 (1H, dd, *J* = 11.7, 4.9 Hz), 5.16 (1H, dd, *J* = 12.7, 7.6 Hz), 2.78 (1H, dd, *J* = 12.9, 3.6 Hz), 2.51 (1H, dd, *J* = 13.4, 5.1 Hz), 2.37 (1H, ddd, *J* = 13.3, 7.8, 3.9 Hz), 2.30 (1H, t, *J* = 12.6 Hz), 2.20 (1H, br s), 2.20 – 2.16 (1H, m), 2.17 (3H, s), 2.09 (1H, dd, *J* = 11.2, 2.6 Hz), 1.98 (1H, dd, *J* = 10.3, 2.9 Hz), 1.74 – 1.54 (3H, m), 1.46 (3H, s), 1.14 (3H, s);
^{13}C NMR (CDCl$_3$): δ 201.8, 175.6, 171.2, 170.0, 143.7, 139.4, 125.1, 108.3, 74.9, 72.1, 64.0, 53.3, 51.3, 43.3, 42.0, 38.1, 35.5, 30.6, 20.6, 18.1, 16.4, 15.2;
HRMS(ESI): $[M+H]^+$ *m/z* 419.1720 (calcd for $C_{22}H_{26}O_8$, 419.1706).

8-*epi*-*O*-Demethylsalvinorin A (4b). InChI=1/C22H26O8/c1-11(23)29-15-8-14(19(25)26)21(2)6-4-13-20(27)30-16(12-5-7-28-10-12)9-22(13,3)18(21)17(15)24/h5,7,10,13-16,18H,4,6,8-9H2,1-3H3,(H,25,26)/t13-,14+,15+,16+,18+,21+,22+/m1/s1/f/h25H
TLC (EtOAc): hR_f = 42, blue to vanillin/H_2SO_4;
^{1}H NMR (CDCl$_3$): δ 7.44 (1H, dt, *J* = 1.7, 0.8 Hz), 7.38 (1H, t, *J* = 1.8 Hz), 6.47 (1H, dd, *J* = 1.9, 0.9 Hz), 5.26 (1H, dd, *J* = 12.0, 1.8 Hz), 5.11 (1H, dd, *J* = 12.7, 7.4 Hz), 2.80 (1H, dd, *J* = 13.1, 3.7 Hz), 2.46 (1H, dd, *J* = 4.7, 1.7 Hz), 2.38 (1H, dd, *J* = 15.1, 2.1 Hz), 2.35 (1H, ddd, *J* = 13.2, 7.5, 3.9 Hz), 2.28 – 2.13 (2H, m), 2.27 (1H, br s), 2.15 (3H, s), 2.04 (1H, td, *J* = 13.8, 3.0 Hz), 1.85 (1H, tt, *J* = 14.0, 4.0 Hz), 1.74 (1H, dt, *J* = 13.6, 3.0 Hz), 1.64 (3H, s), 1.50 (1H, dd, *J* = 15.0, 12.1 Hz), 1.10 (3H, s);
^{13}C NMR (CDCl$_3$): δ 202.3, 176.0, 173.6, 169.9, 143.6, 139.7, 123.3, 108.5, 75.1, 70.1, 64.0, 52.6, 48.0, 45.2, 42.2, 34.7, 33.8, 30.5, 24.6, 20.5, 17.6, 15.3;
HRMS(ESI): $[M+NH_4]^+$ *m/z* 436.1971 (calcd for $C_{22}H_{26}O_8$, 436.1971).

Statement of author contributions

Thomas Munro wrote most of the paper and prepared the figures. Katharine Duncan purified the compounds and grew the crystal of **2b**. Richard Staples obtained and analyzed the diffraction data. Wei Xu performed the radioligand binding assays. Lee-Yuan Liu-Chen supervised Xu. Cécile Béguin supervised the work of

Duncan and Munro. William Carlezon wrote the discussion of salvinorin A's therapeutic potential and SAR. Bruce Cohen supervised and coordinated the entire project.

References

1. Bruker (1997). *ASTRO.* Version 5.007. Bruker AXS Inc., Madison, Wisconsin, USA.
2. Bruker (2001). *SMART.* Version 5.625. Bruker AXS Inc., Madison, Wisconsin, USA.
3. Bruker (2001). *SAINT.* Version 6.22. Bruker AXS Inc., Madison, Wisconsin, USA.
4. Sheldrick GM (1997). *SHELXS97.* University of Göttingen, Germany.
5. Sheldrick GM (1997). *SHELXL97.* University of Göttingen, Germany.
6. Farrugia LJ (2005). *ORTEP-3 for Windows.* Version 1.08. University of Glasgow, Scotland.
7. Sheldrick GM, Bruker (1997). *XCIF.* Version 5.1. Bruker AXS Inc., Madison, Wisconsin, USA.
8. Koreeda M, Brown L, Valdés LJJ: *Chem Lett* 1990**:**2015-2018.
9. Harding WW, Tidgewell K, Schmidt M, Shah K, Dersch CM, Snyder J, Parrish D, Deschamps JR, Rothman RB, Prisinzano TE: *Org Lett* 2005, **7:**3017-20. doi: 10.1021/ol0510522
10. Tidgewell K, Harding WW, Lozama A, Cobb H, Shah K, Kannan P, Dersch CM, Parrish D, Deschamps JR, Rothman RB, Prisinzano TE: *J Nat Prod* 2006, **69:**914-918. doi: 10.1021/np060094b
11. Wang Y, Tang K, Inan S, Siebert DJ, Holzgrabe U, Lee DYW, Huang P, Li J-G, Cowan A, Liu-Chen L-Y: *J Pharmacol Exp Ther* 2005, **312:**220-230. doi: 10.1124/jpet.104.073668
12. Munro TA, Rizzacasa MA: *J Nat Prod* 2003, **66:**703-705. doi: 10.1021/np0205699
13. Tidgewell K, Harding WW, Schmidt M, Holden KG, Murry DJ, Prisinzano TE: *Bioorg Med Chem Lett* 2004, **14:**5099-5102. doi: 10.1016/j.bmcl.2004.07.081
14. Munro TA. *PhD Thesis*. University of Melbourne 2006. [http://eprints.infodiv.unimelb.edu.au/archive/00002327]
15. Béguin C, Carlezon W, Cohen BM, He M, Lee DY-W, Richards MR, Liu-Chen L-Y: *Salvinorin derivatives and uses thereof.* US Patent Application 20060052439; 2006.

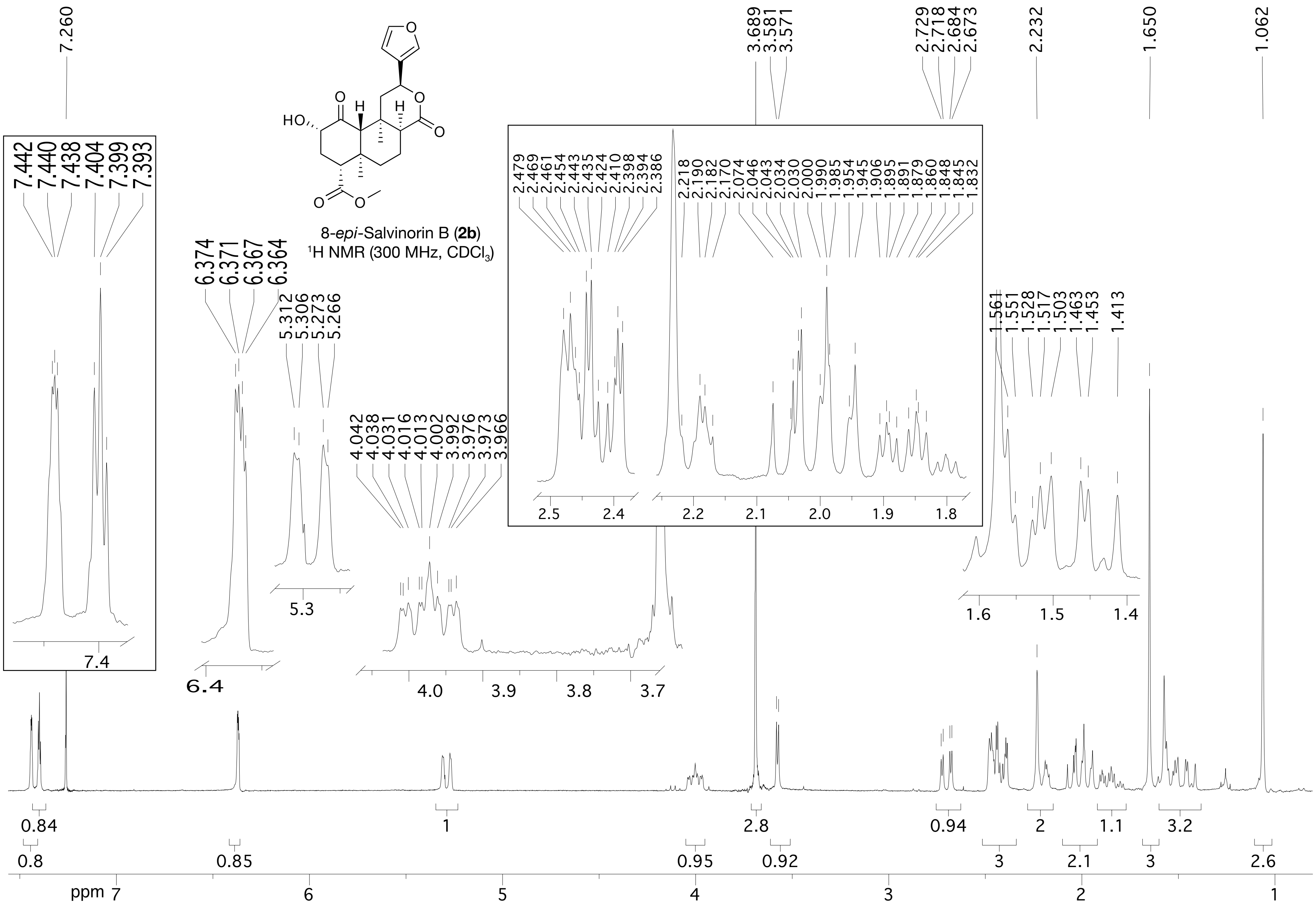

8-*epi*-Salvinorin B (**2b**)
^{1}H NMR (300 MHz, $CDCl_3$)

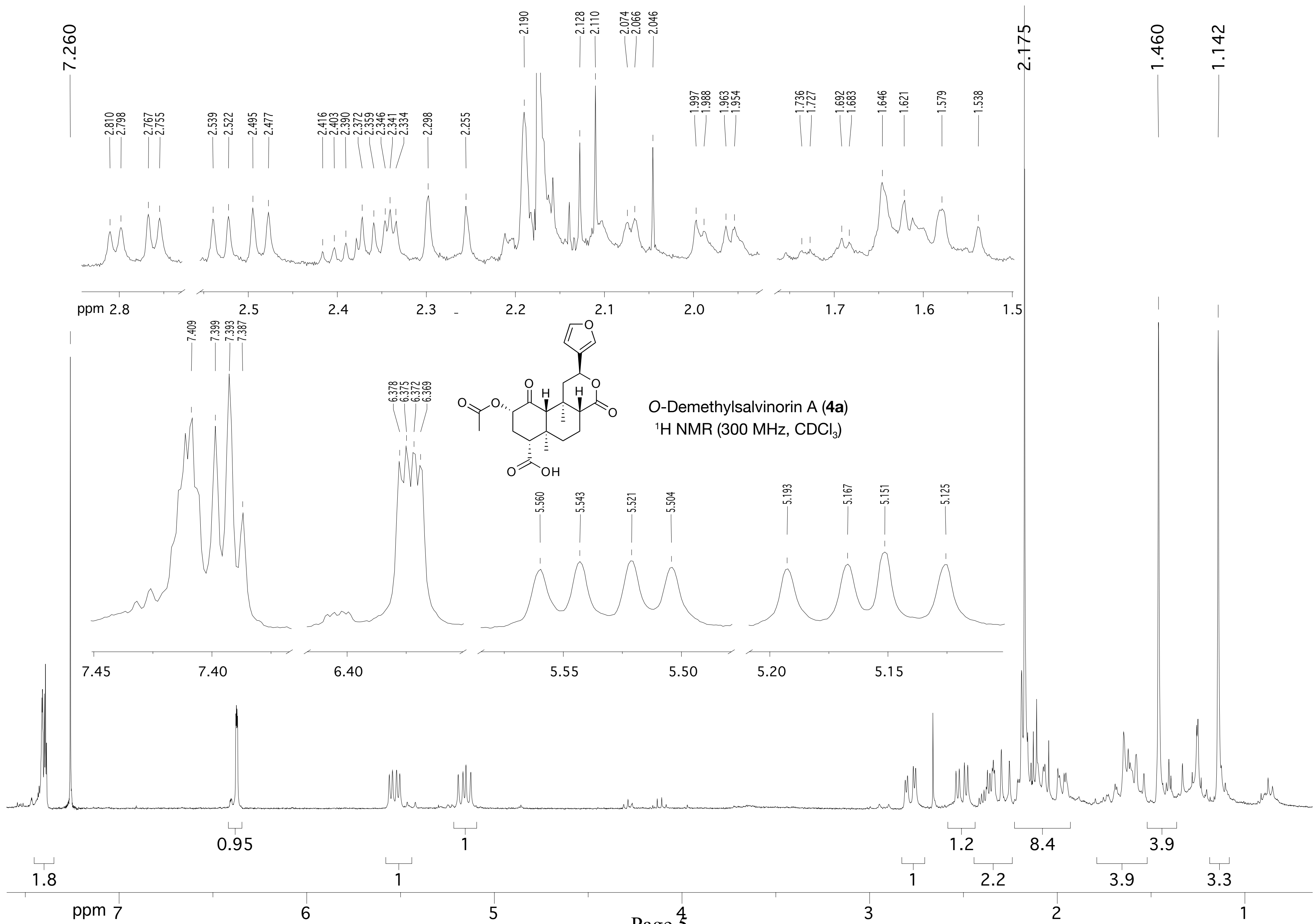
O-Demethylsalvinorin A (4a)
1H NMR (300 MHz, CDCl3)
7.260
2.175
1.460
1.142
2.810 2.798 2.767 2.755 2.539 2.522 2.495 2.477 2.416 2.403 2.390 2.372 2.359 2.346 2.341 2.334 2.298 2.255
2.190 2.128 2.110 2.074 2.066 2.046 1.997 1.988 1.963 1.954
1.736 1.727 1.692 1.683 1.646 1.621 1.579 1.538
7.409 7.399 7.393 7.387
6.378 6.375 6.372 6.369
5.560 5.543 5.521 5.504
5.193 5.167 5.151 5.125
0.95
1
1.2
8.4
3.9
1.8
1
1
2.2
3.9
3.3
ppm

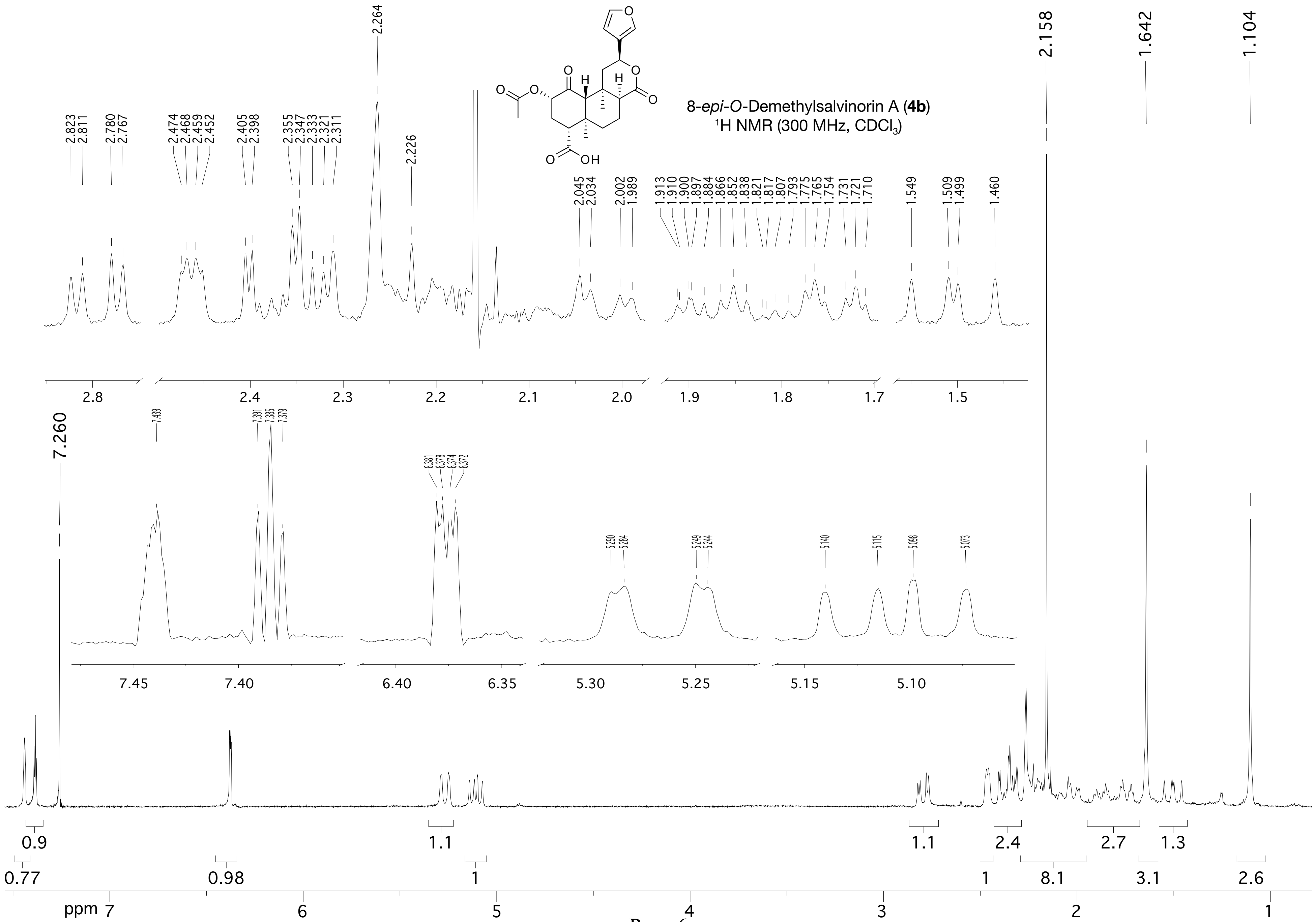
8-*epi*-*O*-Demethylsalvinorin A (**4b**)
^{1}H NMR (300 MHz, $CDCl_3$)
ppm
